# Producing and evaluating a novel lentiviral vector for β-thalassaemia gene therapy

**DOI:** 10.1186/1753-6561-6-S4-O15

**Published:** 2012-07-09

**Authors:** Y Bokinni, V Kao, M García-Gómez, M Antoniou

**Affiliations:** 1 King’s College London, School of Medicine, Department of Medical and Molecular Genetics, UK

## Introduction

The β-haemoglobinopathies are of the most prevalent inherited disorders worldwide. β-thalassaemia is a single gene disorder affecting the β-globin gene, thus resulting in a lack or depleted availability of β -globin for formation of haemoglobin. β-thalassaemia has become a target for gene therapy based treatments in hope of a cure, or significant phenotype amelioration. The technique aims to treat the haematopoietic stem cells (HSC) of patients with the viral vectors *ex vivo*, in the hope of significant β -globin mRNA transcript production on HSC erythroid differentiation post re-transplantation. Numerous investigations have been conducted in the use of Lentiviral vectors harbouring human β-globin transcription units, only one of which has proceeded into clinical trials (Cavazzana-Calvo, Payen et al. 2010). The ultimate aim of all 'construct' designs is to present significant phenotype amelioration with an average of one vector copy number (VCN) per HSC.

## Methods

Antoniou's group have recently devised a number of “GLOBE” constructs based on previous functional studies with the inclusion of regions physiologically present within the endogenous β -globin gene, previously deemed insignificant, and therefore, omitted from to all known published constructs to date. Previous observations with gammaretroviral vectors had determined the inclusion of the full β-globin 2nd intron to be highly detrimental to viral production and quality (Leblouch, 1994). Based on recent findings, the inclusion of a transcriptional terminator region and full 2nd intron have been added, yielding the latest generation of Lentiviral vector constructs (GLOBE-2 and GLOBE-4) .The aim of this project was to produce these Lentiviral vectors and conduct a comparative expression analysis via transducing Murine erythroleukaemia cells.

## Results and conclusions

Average viral titres obtained for the GLOBE-2 and GLOBE-4 constructs were 7.2x107 and 5x107 viral particles (vp)/ml respectively, incurring a 31% variance despite a 600bp difference in size. The relative amounts of β-globin expression adjusted to level of expression per vector copy were 0.869 (± 0.21) and 0.061 (±0.07) for GLOBE 4 and 2, thus revealing greater levels of expression for our novel GLOBE 4 construct. The results obtained suggest that LV production is not severely negatively affected by the inclusion of the full 2nd intron (a finding not published to date), and that its presence may indeed significantly increase mature human β-globin mRNA levels.

